# Ultra-stable clock laser system development towards space applications

**DOI:** 10.1038/srep33973

**Published:** 2016-09-26

**Authors:** Dariusz Świerad, Sebastian Häfner, Stefan Vogt, Bertrand Venon, David Holleville, Sébastien Bize, André Kulosa, Sebastian Bode, Yeshpal Singh, Kai Bongs, Ernst Maria Rasel, Jérôme Lodewyck, Rodolphe Le Targat, Christian Lisdat, Uwe Sterr

**Affiliations:** 1 School of Physics and Astronomy, The University of Birmingham, Birmingham B15 2TT, United Kingdom; 2 Physikalisch-Technische Bundesanstalt (PTB), Bundesallee 100, 38116, Braunschweig, Germany; 3 LNE-SYRTE, Observatoire de Paris, PSL Research University, CNRS, Sorbonne Universités, UPMC Univ. Paris 06, 61 Avenue de l’Observatoire, 75014 Paris, France; 4 Institute of Quantum Optics, Leibniz Universität Hannover, Welfengarten 1, D-30167 Hannover, Germany

## Abstract

The increasing performance of optical lattice clocks has made them attractive for scientific applications in space and thus has pushed the development of their components including the interrogation lasers of the clock transitions towards being suitable for space, which amongst others requires making them more power efficient, radiation hardened, smaller, lighter as well as more mechanically stable. Here we present the development towards a space-compatible interrogation laser system for a strontium lattice clock constructed within the Space Optical Clock (SOC2) project where we have concentrated on mechanical rigidity and size. The laser reaches a fractional frequency instability of 7.9 × 10^−16^ at 300 ms averaging time. The laser system uses a single extended cavity diode laser that gives enough power for interrogating the atoms, frequency comparison by a frequency comb and diagnostics. It includes fibre link stabilisation to the atomic package and to the comb. The optics module containing the laser has dimensions 60 × 45 × 8 cm^3^; and the ultra-stable reference cavity used for frequency stabilisation with its vacuum system takes 30 × 30 × 30 cm^3^. The acceleration sensitivities in three orthogonal directions of the cavity are 3.6 × 10^−10^/g, 5.8 × 10^−10^/g and 3.1 × 10^−10^/g, where g ≈ 9.8 m/s^2^ is the standard gravitational acceleration.

Narrow linewidth lasers with ultra-high stability are in great demand for many fields of science like fundamental physics^1^,^2^ (e.g. test of Lorentz invariance^3^ or Einstein’s theory of General Relativity^4^), metrology^5^,^6^, communication^7^ and astrophysics^8^. With optical clocks in space, test of the gravitational red shift, of the Einstein Equivalence principle^9^ at a much higher level of accuracy as compared to ground, and relativistic geodesy can be widely applied by observing the gravitational potential of a portable ground clock in comparison to a master clock in space^10^.

Recent technological developments of optical lattice clocks have pushed the boundaries towards making them not only more robust but also smaller and more portable^11^,^12^. As part of that development there is a need to make a narrow linewidth laser for spectroscopy of the clock transition that follows this trends. The most important part of such a laser is a reference cavity that defines the stability of the laser as well as its linewidth. To achieve this, the cavity should be insensitive to the external factors such as vibrations or temperature fluctuations. Some efforts have been made in this direction with new designs targeting force-insensitivity^13^,^14^,^15^,^16^, compactness and transportability^17^ and space readiness^18^. The importance of such lasers was boosted by European Space Agency (ESA), which has launched the Space Optical Clock (SOC) projects, developing next generations of mobile lattice clocks towards space applications. Strontium was chosen for both SOC and SOC2 iterations of the project^11^,^19^,^20^ and thus it was necessary to develop the clock laser operating specifically at the frequency of the 429-THz clock transition in strontium.

The clock laser is used for the interrogation of the clock transition (natural linewidth 1.2 mHz^21^) and therefore it needs to have an intrinsic narrow linewidth. This ultra-high stability of the laser’s frequency is typically obtained by stabilising it to a high finesse Fabry-Pérot cavity. As the fractional frequency stability is equal to the fractional length stability of the optical cavity, the cavity mirrors are rigidly separated by a highly length-stable spacer, made for example of ultra-low expansion glass (ULE), making it less sensitive to temperature instabilities. In addition, thermodynamic fluctuations of the mirrors fundamentally limit the cavity stability^22^, with the Brownian thermal noise being most significant, which sets additional requirements on the material of the spacer, the mirror substrates and the mirror coatings. Moreover, the cavity should be as insensitive as possible to external vibrations that would change the distance between the mirrors.

In this paper, we report on the design and characteristics of a clock laser system, which is the next iteration in the pursuit of space applications. To our knowledge, this is the first clock laser system for a strontium optical lattice clock, where the reference cavity is based on a design by space industry, presented by Argence *et al*.^18^. The cavity features an optimised size, rigid construction that is designed to withstand large rotations and vibrations, e.g. during launch, as well as reduced power consumption. In this paper we discuss the sensitivity of the cavity to accelerations, laser power sensitivity and present the overall stability of the reference resonator. In addition, we report on the construction of a compact clock laser system that is powered with a single diode laser that provides sufficient power via optical fibres for frequency stabilisation to the optical cavity, atom interrogation of the clock transition (429 THz ^1^*S*_0_ − ^3^*P*_0_) and optical frequency comb referencing. A fibre length stabilisation is included on latter two, while it was not found necessary to stabilise the short and well protected fibre link to the cavity.

## Results

### Stability

**Figure 1:**
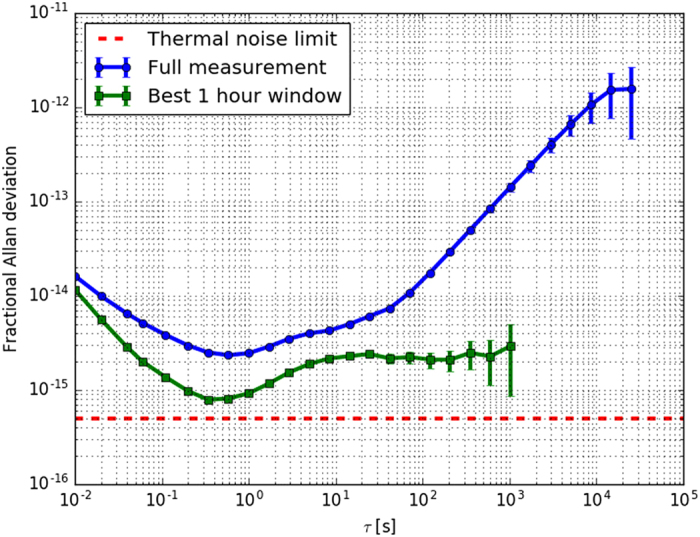
Fractional Allan deviation (linear drift removed) of a beat note against stationary system. Blue circles indicate a full 24 h measurement with a minimum instability of 2.5 × 10^−15^ in 1 s. The best 1 hour slot within the 24 hour measurement is plotted with green squares with a minimum of 7.9 × 10^−16^ in less than 1 s. The estimated thermal noise limit for the cavity is 5 × 10^−16^, as presented by red-dashed line.

We employ a 10 cm long ULE cavity with thermally compensated fused silica mirrors (please refer to ‘Methods → Optical cavity’ section for details), leading to an estimated thermal noise fractional frequency instability level of 5 × 10^−16^. The stability of the laser was assessed by comparing it to a reference stationary clock laser system, based on an ultra-stable long cavity, that was operating at the same frequency. This reference laser has an instability below 1 × 10^−16^ at averaging times from 1 s to 1000 s and is described in detail by Häfner *et al*.^23^. During an uninterrupted lock period of 24 hours, the beat note was recorded with a deadtime-free frequency counter. Because the reference laser has an instability well below the laser under test, the instability of the measured beat note between the two lasers completely reflects the stability of the examined laser. The frequency data was processed by removing the linear drift first, as it can be easily compensated for using an acousto optical modulator (AOM) in the working clock setup. To express the fractional laser frequency instability we use the Allan deviation, which reached 2.5 × 10^−15^ in 1 s for the 24 hour measurement (Fig. 1 blue circles). To assess the smallest achieved instability of the laser, a script was written to find the most stable 1 hour slot in the 24 hour measurement. The green squares in Fig. 1 present Allan deviation for the best 1 hour slot, which reaches a minimum of 7.9 × 10^−16^ in less than 1 s, close to the estimated thermal noise limit of 5 × 10^−16^. Thanks to the reference laser being part of a working strontium lattice clock, we could assess a long-term drift of the laser frequency under test to be 0.1 Hz/s over a period of nine months due to ageing of the spacer material and up to 0.4 Hz/s within 100 s, most likely from the temperature drifts or residual pressure fluctuations.

### Acceleration sensitivity

**Figure 2:**
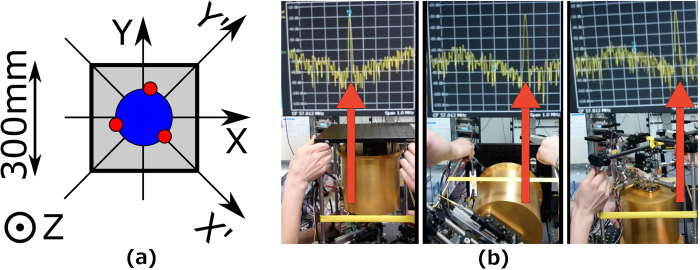
**(a)** Coordinates assigned to the cavity (top view). Grey colour is the breadboard on which the cavity vacuum chamber stands, the blue circle shows the body of the cavity and the red circles indicate the positions where the cavity is supported (please refer to ‘Methods → Optical cavity’ section for details). **(b)** Figure presenting the procedure of measuring the acceleration sensitivity along Z direction. The laser stayed locked to the cavity while the resonator was turned by 180° along X axis. The beat note against stationary system was recorded with a frequency counter and is visible on a spectrum analyser on top of each frame with a red arrow pointing at the peak. The span of the spectrum analyser is 1 MHz.

We measured the influence of accelerations on the resonance frequency of the cavity by recording the beat note while performing a set of rotations to align the different axes with the gravitational acceleration vector, effectively changing the acceleration by the gravitational acceleration *g* = 9.8 m/s^2^. A coordinate system assigned to the cavity is presented in Fig. 2(a). A rotation performed to measure acceleration sensitivity along the Z axis is presented in Fig. 2(b).

To measure the sensitivity along the Z axis, the cavity was turned upside down to change the acceleration value along the axis from −g to g leaving the acceleration along the other axes unchanged. The value of the acceleration sensitivity was calculated from the observed frequency shift of the beat note to be −3.6 × 10^−10^/g in fractional frequency units.

With the value of Z acceleration sensitivity known, X and Y directions were measured rotating the resonator by 90° so that the acceleration along the Z axis becomes zero while it equals *g* along the other axis. We found an acceleration sensitivity along the X axis equal to −5.8 × 10^−10^/g and along the Y axis equal to −3.1 × 10^−10^/g.

As mechanical nonlinearities in the cavity mount may influence these large-acceleration results, we did additional measurements for small tilts along axes X′ and Y′, which are rotated by 45° around the Z axis. The tilt angle was ±2.7° that gave the sensitivity values of −2.0 × 10^−11^/g for X′ and −3.5 × 10^−10^/g for Y′, being more appropriate for the small accelerations during operation. Because of the small sensitivity along X′, a value for the vertical sensitivity Z could also be obtained from the observed quadratic dependency of the frequency on the tilt, which is in good agreement with the value obtained from flipping the cavity and equal to −2.4 × 10^−10^/g. The cavity is mounted by three-point support fixing, which provides different symmetry for perpendicular axes. For a perfect three-fold symmetry of the cavity and its mounting, zero sensitivity in all directions would be expected. Thus, we attribute the observed difference in sensitivity for X and Y direction to tolerances and imperfections in the mechanical setup, and the geometry of the cavity.

### Power sensitivity

**Figure 3:**
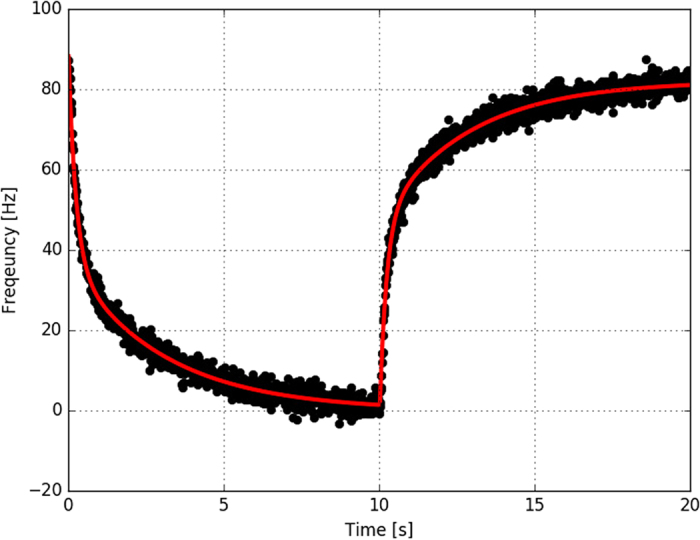
Averaged change in the optical frequency of the clock laser as a result of power modulation of the light injected to the cavity. Initially the power on the input was reduced for 10 s by Δ*P* = 0.5 *μ*W with a square modulation as measured on the cavity transmitted power. The red line shows a double exponential *f*(*t*) = Δ*P*(*A*_1_ exp(−*t*/*τ*_1_) + *A*_2_ exp(−*t*/*τ*_2_)) + *C* fitted to the drop and the rise. Parameters obtained from the fit are *A*_1_ = −72 Hz/*μ*W, *A*_2_ = −104 Hz/*μ*W, *τ*_1_ = 2.9 s and *τ*_2_ = 0.23 s.

Laser power fluctuations cause changes in the clock laser’s frequency due to the light being absorbed by the mirrors. The absorbed power causes heating and deformation of the mirror coating and substrate, leading to distance change between the mirrors. We investigated the effect using the same ultra-stable stationary clock laser system as a reference. We applied a square modulation to the amplitude of the laser power incident on the cavity, while measuring the beat note against the stationary system. The modulation amplitude was Δ*P* = 0.5 *μ*W, measured on the transmitted laser power and the modulation period was 20 s. As the timescales under consideration are much larger than the photon lifetime inside the cavity (*τ*_*RD*_ ~ 25 *μ*s), we can assume that the power inside the cavity instantaneously follows the incident power. The averaged beat note frequency with the linear drift removed is presented in Fig. 3. The red curve is a double exponential fit *f*(*t*) = Δ*P*(*A*_1_ exp(−*t*/*τ*_1_) + *A*_2_ exp(−*t*/*τ*_2_)) + *C*. The value of the power sensitivity was calculated from the asymptote of the fit and is equal to 180 Hz/*μ*W or 4.2 × 10^−13^/*μ*W in fractional frequency units. The fitted double exponential function gives us both a long time constant (*τ*_1_ = 2.9 s) and a short one (*τ*_2_ = 0.23 s). For the sensitivities, we find *A*_1_ = −72 Hz/*μ*W for the long-term effect and *A*_2_ = −104 Hz/*μ*W for the short-term. We believe that the short and long-term exponentials represent thermal diffusion across the cavity-mode diameter and across the free standing mirror diameter respectively, which was studied with respect to the photo-thermal effect by Farsi *et al*.^24^.

**Figure 4:**
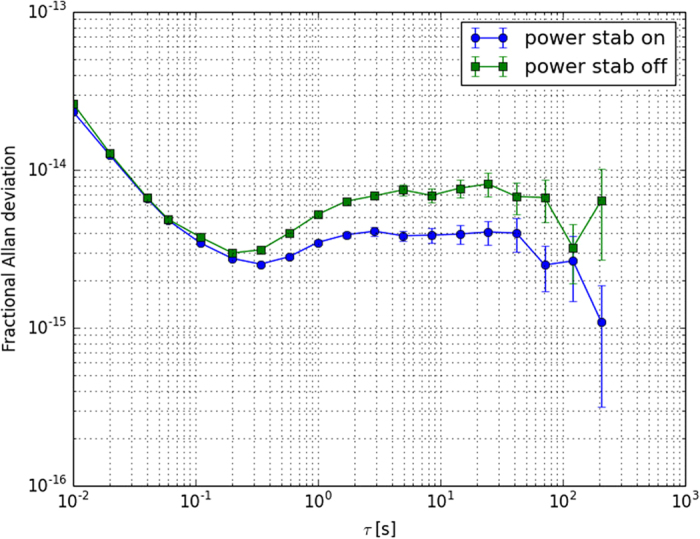
Fractional Allan deviation of the laser under test with power stabilisation on (blue circles) and power stabilisation off (green squares). As the cavity’s resonance frequency is affected by power fluctuations of the injected light due to photo-thermal effect, the stability is improved by active stabilisation of the power transmitted by the cavity.

To reach a 10^−15^ fractional frequency instability level the transmitted power of 1.5 *μ*W has to be stable at a level of approximately 3.5 nW, or 2.3 × 10^−3^ in relative power. Experimentally, we found large power fluctuations due to both the spatial mode filtering with a fibre and the laser amplitude fluctuations. To reduce this effect we stabilise the transmitted power by using a double-pass AOM. The Allan deviations with and without power stabilisation are presented in Fig. 4 and show slight improvement for averaging times *τ* bigger than 100 ms. This reduction is much less than what is expected from the in-loop reduction of the power fluctuations. We attribute the difference to relatively high pressure level inside the vacuum chamber (2.5 × 10^−6 ^mbar) or additional fluctuations appearing between the cavity and the photodetector, from air currents and spurious interferences.

### Pressure sensitivity

**Figure 5:**
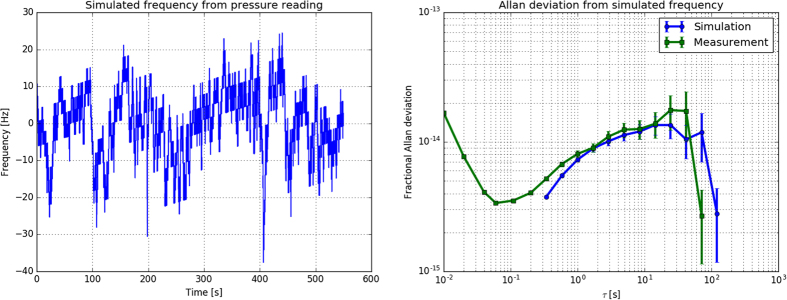
(left) Simulation of frequency fluctuations based on measured pressure fluctuations inside the cavity’s vacuum chamber and the corresponding fractional Allan deviation (right, blue circles) with linear drifts removed. The frequency was simulated assuming the refractive index of air for the residual gas of (*n*−1)/*p* = 2.7 × 10^−7^/mbar. **(right)** The green squared line shows the fractional Allan deviation of a measured frequency of the laser that was taken on the same day as the pressure measurement.

During the measurement, the pressure inside the vacuum chamber stayed at a level of 2.5 × 10^−6^ mbar with about 10% fractional fluctuations. Using the modified Edlén formula for the refractive index of air^25^, we estimate the refractivity of air at a pressure of 1 mbar to be at *n* − 1 = 2.7 × 10^−7^. The calculated pressure sensitivity at this pressure level is −120 Hz/10^−6^ mbar or −3 × 10^−13^/10^−6^ mbar in fractional frequency units. To see how the pressure fluctuations affect the frequency measurement, we have recorded the pressure inferred from the ion pump current. From the recorded data, we have calculated the corresponding frequency fluctuations and the Allan deviation (Fig. 5). The calculated Allan deviation is compared with the Allan deviation of a measured laser frequency in the right panel of the Fig. 5. The calculations indicate that pressure changes may be the limiting factor for the stability of our setup. During the periods of lower frequency instability, the vacuum pressure was probably more stable or at a lower level. The relatively high pressure level is caused by leaks on the flange of the vacuum chamber that will be fixed in the near future.

## Discussion

Overall, the laser shows a sufficiently good performance with an instability below 10^−15^ with a low long-term drift and it can be successfully used as a part of an optical lattice clock. The advantage of the laser is its modular construction and stabilised fibre-link ports embedded in the laser distribution module, which keep the transferred optical frequency stable between the modules despite the acoustic noise and temperature fluctuations. The reference cavity also features a reliable fixing mount that is believed to withstand rocket launch and yet is more cost effective compared with its archetype^18^. The whole setup is designed to lower the power consumption by using components that are temperature stabilised closely to room temperature.

The pressure inside the vacuum chamber seems to have the largest influence on the stability of our setup and in future, it must be improved in order to operate the clock. As long as the pressure is at the 10^−6 ^mbar level, its fluctuations will have a dominant influence on the stability of the laser. By lowering the pressure down to 10^−7 ^mbar these contributions should drop below the thermal noise limit. Although the previous SOC iteration of the clock laser had slightly better stability^14^, we believe that our setup can be improved beyond that level by fixing the leaks in the cavity vacuum chamber.

The acceleration sensitivity is of the order of 3 × 10^−10^/g for its upright position, which is one order of magnitude higher than in the original design presented by Argence *et al*.^18^ (4 × 10^−11^/g) or the alternate designs presented by Nazarova *et al*.^13^ (3.3 × 10^−11^/g) and Webster *et al*.^15^ (2.5 × 10^−11^/g). The current design might be more sensitive to acceleration because of the fixing where e.g. the inner heat shield is connected to the cavity mounting ring. It is believed that the acceleration sensitivity might be improved by one order of magnitude by improving the mounting. Additionally, it is possible to compensate for the frequency change caused by acceleration, by applying an active feed-forward that was presented by Leibrandt *et al*.^16^. The large microvibrations on the International Space Station (ISS) are on the level of mg/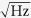 around one Hz, but they can be reduced to the *μ*g/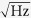 level by active isolation like the Microgravity Vibration Isolation Subsystem (MVIS) that is currently used on the ISS^26^,^27^. Thus the thermal limit of the laser stability of 5 × 10^−16^ can be reliably reached. In addition, with low thermal noise crystalline mirrors that are now becoming available^28^, for the same cavity size stabilities of 2 × 10^−16^ are possible, less than a factor of three away from the best stationary laser systems. Already with the current laser stability, similar to results from stationary clocks^29^, a clock instability in the range of 10^−14^ at one second averaging time is expected.

## Methods

### Laser head

The laser head that is used in the setup is a fibre-coupled interference-filter-stabilised external-cavity diode laser (ECDL)^30^ that was developed for compactness and robustness. The 10 cm long external resonator provides an intrinsic narrow fast laser linewidth of less than 10 kHz (free-running, Lorentzian linewidth). With a wavelength-selected AR-coated laser diode the strontium clock laser can be operated with diode-laser temperature close to room temperature while delivering 15 mW power at the fibre output.

**Figure 6:**
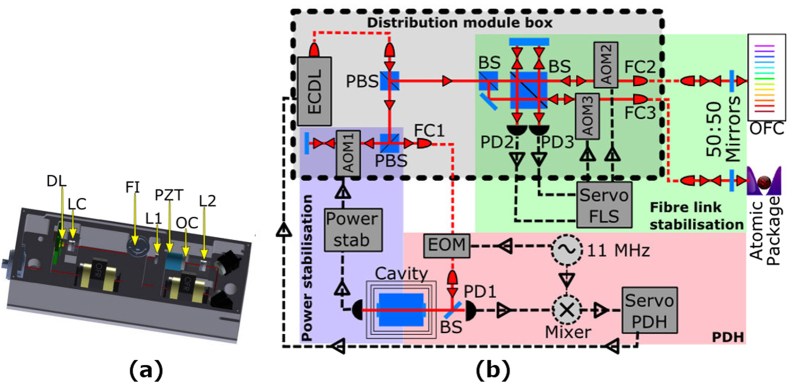
**(a)**Drawing of the external cavity diode laser (ECDL) with an interference filter (FI) for wavelength selection. (DL–laser diode, LC–collimating lens, L1–focusing lens, OC–output coupler, PZT–piezo-electric transducer, L2–re-collimating lens). **(b)** Overview of the distribution module. Light from the ECDL is coupled into a fibre for spatial mode filtering and split at the output for three different beams. One beam after double-passing an acousto optical modulator (AOM1) is sent through a fibre-coupled electro optical modulator (EOM) to provide frequency modulation sidebands for the Pound-Drever-Hall (PDH) locking. The second beam passes through AOM2 and is coupled into a fibre for reference with an optical frequency comb (OFC). The third beam passes through AOM3 and is coupled into a fibre that will transfer light into the atomic package. After both fibres there are half-reflecting mirrors that couple part of the light back into the fibres. Photo diodes (PD2 and PD3) are used to detect the beat note of the reflected light with the original beam before the AOM’s. The beat note signal is sent to frequency length stabilisation (FLS) servo that compensates for phase fluctuations in the fibre link with an AOM.

The laser design is presented in Fig. 6(a) and consists of an aspheric lens with *f* = 3.1 mm that collimates the output beam of the laser diode. Another aspheric lens with *f* = 18.4 mm is used as a cat’s eye to focus the beam onto the out-coupling mirror that is glued to a piezo stack. Inside the laser cavity, a 1 nm FWHM (Full Width at Half Maximum) interference filter on a rotation mount provides laser wavelength selection. A third lens, which is identical to the one of the cat’s eye, recollimates the laser output beam. The reflectivity of the out-coupling mirror is 30%. For temperature stabilisation of the laser diode we use a Peltier element. The laser housing is also stabilised to a few degrees Celsius above room temperature using additional Peltier elements between the laser housing and the base plate. The laser output light is fed through a 40 dB optical isolator and coupled into a polarisation-maintaining single-mode fibre with FC/APC connector that acts as a mode filter to provide a filtered Gaussian-shaped beam to the distribution module.

### Distribution module

Inside the distribution module, the light from the fibre is collimated and split into three branches as shown in Fig. 6(b). The first branch passes through a double-pass AOM and then through a fibre-coupled EOM, and is used for locking to the high-finesse cavity with the Pound-Drever-Hall method. The second beam passes through an AOM into a fibre that connects to the atomic package for the clock spectroscopy. In the present investigations, this output was used to compare the laser with a reference laser for characterisation. After the fibre, a small amount of light is reflected back into the fibre. The reflected beam passes once again through the AOM and then it is superimposed with a reference beam before the fibre. The resulting beat note is used to compensate for any phase-shifts that arose in the fibre. The third beam is sent through a fibre to the optical frequency comb. That fibre is similarly compensated with its own AOM. The same reference beam splitter and the same reference mirror are used for the two fibre noise cancellations to avoid any out-of-loop paths and corresponding frequency offsets between atoms and comb.

### Optical cavity

**Figure 7:**
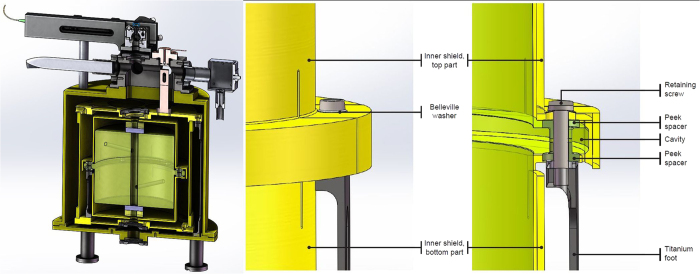
(left) Cut through the vacuum system containing the ultra-high finesse optical cavity used to stabilise the clock laser. The spacer of the cavity is made from Ultra Low Expansion (ULE) glass to which fused silica mirrors with thermal compensation rings are optically contacted. The cavity is mounted on three titanium legs and surrounded by two gold plated aluminium shields and the gold plated aluminium vacuum chamber. The outer heat shield is connected to a temperature-stabilised copper thermal feedthrough, which is temperature stabilised with a thermoelectric cooler. The vacuum chamber is pumped by a 2 l/s ion pump and a non-evaporable getter. Laser beam is coupled from the top and the transmitted signal leaves the cavity through the bottom viewport. **(center)** Details of the mounting of the cavity with view on the inner heat shield. **(right)** Detailed cut of the mounting of the cavity and the inner heat shield to the titanium legs.

The ultra-stable cavity was designed with the goal of minimising the thermal noise, vibration sensitivity and temperature sensitivity. As presented in Fig. 7, the resonator is oriented vertically, which has the advantage of significantly reducing the influence of seismic noise along this direction^31^. Halfway along the spacer is a cylindrical shoulder with a length of 7 mm for mounting. The cavity is mounted on this central shoulder by screws with Belleville washers on three thin titanium legs^32^ leading to a configuration where the cavity is rigidly constrained in the vertical direction. The three legs are flexible only in the radial direction, which avoids radial stress and corresponding deformations on the cavity while still providing a kinematically rigid mount. From the fact that the coupling to the cavity did not change significantly, when the setup was rotated by 90° we estimate a gravitational sag of less than 20 *μ*m and thus a first mechanical resonance frequency for radial motion of more than 100 Hz. The spacer is made of ULE glass with a 110 mm diameter. Finite element simulations show that a horizontal, x-y plane acceleration mainly induces a tilt of the cavity mirrors, with corresponding vibration sensitivity coefficients 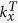 and 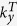 that are non-zero if the optical axis and the mechanical axis are not perfectly aligned. Both the magnitude and sign of the tilt depend on the aspect ratio (i.e. length/diameter) of the cavity. A spacer length *L* = 100 mm was chosen as it minimises the mirror tilt from horizontal accelerations^31^. Ideally, the vertical vibration sensitivity 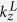 should be zero from symmetry considerations, as the forces on the shoulder act from the top and bottom surface on the symmetrically located mounting ring.

The resonator is formed by a flat mirror and a mirror with a 1 m radius of curvature, which leads to respective 1/*e*^2^ mode radii of 258 *μ*m and 272 *μ*m at 698 nm. The dielectric coating of the mirrors is deposited on fused silica substrates in order to reduce the contribution to the thermal noise. For the assembly, the corresponding laser instability is estimated to be around 5 × 10^−16^. The mismatch between the CTE (Coefficients of Thermal Expansion) of the spacer (ULE) and the mirrors substrates (Fused Silica) must be compensated for, to avoid degrading the temperature insensitivity of the cavity. For this purpose, an ULE annulus is optically contacted to the back of each mirror to limit the expansion of the substrates^33^. Additionally, the influence of external temperature fluctuations is reduced by enclosing the cavity in three aluminium shields that are plated with gold in order to minimise their emissivity. The outermost shield forms the vacuum chamber, which is pumped by a 2 l/s ion pump and a non-evaporable getter that are designed to maintain the vacuum in the 10^−7 ^mbar range. This pressure is limited by the outgassing rates of the employed materials. The intermediate shield is temperature-regulated at the level of millikelvin by a copper finger and a Peltier element.

The vacuum chamber with the cavity inside is placed on an active isolation platform to minimise the influence of the ambient vibrational noise.

## Additional Information

**How to cite this article**: Świerad, D. *et al*. Ultra-stable clock laser system development towards space applications. *Sci. Rep*. **6**, 33973; doi: 10.1038/srep33973 (2016).

## References

[b1] Ludlow A. D., Boyd M. M., Ye J., Peik E. & Schmidt P. O. Optical atomic clocks. Reviews of Modern Physics 87, 637–701, 10.1103/RevModPhys.87.637 (2015).

[b2] Bize S. . Cold atom clocks and applications. Journal of Physics B: Atomic, Molecular and Optical Physics 38, S449–S468, 10.1088/0953-4075/38/9/002 (2005).

[b3] Eisele C., Nevsky A. Y. & Schiller S. Laboratory test of the isotropy of light propagation at the 10^−17^ level. Physical Review Letters 103, 1–4, 10.1103/PhysRevLett.103.090401 (2009). 19792767

[b4] Chou C. W., Hume D. B., Rosenband T. & Wineland D. J. Optical clocks and relativity. Science 329, 1630–1633, 10.1126/science.1192720 (2010). 20929843

[b5] Katori H. Optical lattice clocks and quantum metrology. Nature Photonics 5, 203–210, 10.1038/nphoton.2011.45 (2011).

[b6] Cygan A. . Cavity mode-width spectroscopy with widely tunable ultra narrow laser. Optics Express 21, 29744, 10.1364/OE.21.029744 (2013). 24514525

[b7] Al-Taiy H., Wenzel N., Preußler S., Klinger J. & Schneider T. Ultra-narrow linewidth, stable and tunable laser source for optical communication systems and spectroscopy. Optics Letters 39, 5826–9, 10.1364/OL.39.005826 (2014). 25361095

[b8] Abbott B. P. . LIGO: the Laser Interferometer Gravitational-Wave Observatory. Reports on Progress in Physics 72, 076901, 10.1088/0034-4885/72/7/076901 (2009).

[b9] Altschul B. . Quantum tests of the Einstein equivalence principle with the STE-QUEST space mission. Advances in Space Research 55, 501–524, 10.1016/j.asr.2014.07.014 (2015).

[b10] Bjerhammar A. On a relativistic geodesy. Bulletin Géodésique 59, 207–220, 10.1007/BF02520327 (1985).

[b11] Bongs K. . Development of a strontium optical lattice clock for the SOC mission on the ISS. Comptes Rendus Physique 16, 553–564, 10.1016/j.crhy.2015.03.009 (2015).

[b12] Poli N. . A transportable strontium optical lattice clock. Applied Physics B: Lasers and Optics 117, 1107–1116, 10.1007/s00340-014-5932-9 (2014).

[b13] Nazarova T., Riehle F. & Sterr U. Vibration-insensitive reference cavity for an ultra-narrow-linewidth laser. Applied Physics B: Lasers and Optics 83, 531–536, 10.1007/s00340-006-2225-y (2006).

[b14] Vogt S. . Demonstration of a transportable 1 Hz-linewidth laser. Applied Physics B: Lasers and Optics 104, 741–745, 10.1007/s00340-011-4652-7 (2011).

[b15] Webster S. & Gill P. Force-insensitive optical cavity. Optics Letters 36, 3572–3574, 10.1364/OL.36.003572 (2011). 21931394

[b16] Leibrandt D. R., Bergquist J. C. & Rosenband T. Cavity-stabilized laser with acceleration sensitivity below 10^−12 ^g^−1^. Phys. Rev. A 87, 023829, 10.1103/PhysRevA.87.023829 (2013).

[b17] Chen Q.-F. . A compact, robust, and transportable ultra-stable laser with a fractional frequency instability of 1 × 10^−15^. The Review of Scientific Instruments 85, 113107, 10.1063/1.4898334 (2014). 25430098

[b18] Argence B. . Prototype of an ultra-stable optical cavity for space applications. Optics Express 20, 25409–20, 10.1364/OE.20.025409 (2012). 23187358

[b19] Schiller S. . The space optical clocks project. In *International Conference on Space Optics (Proceedings)*, 1–5, http://www.exphy.uni-duesseldorf.de/optical_clock/PDF/Schiller (2010).

[b20] Schiller S. . The space optical clocks project: Development of high-performance transportable and breadboard optical clocks and advanced subsystems. In EFTF 2012-2012 European Frequency and Time Forum, Proceedings, 412–418, 10.1109/EFTF.2012.6502414 (2012).

[b21] Porsev S. G., Ludlow A. D., Boyd M. M. & Ye J. Determination of Sr properties for a high-accuracy optical clock. Physical Review A–Atomic, Molecular, and Optical Physics 78, 032508, 10.1103/PhysRevA.78.032508 (2008).

[b22] Kessler T., Legero T. & Sterr U. Thermal noise in optical cavities revisited. Journal of the Optical Society of America B 29, 178, 10.1364/JOSAB.29.000178 (2012).

[b23] Häfner S. . 8 × 10^−17^ Fractional laser frequency instability with a long room-temperature cavity. Optics Letters 40, 2112–2115, 10.1364/OL.40.002112 (2015). 25927798

[b24] Farsi A., Siciliani De Cumis M., Marino F. & Marin F. Photothermal and thermo-refractive effects in high reflectivity mirrors at room and cryogenic temperature. Journal of Applied Physics 111, 043101, 10.1063/1.3684626 (2012).

[b25] Birch K. P. & Downs M. J. Correction to the Updated Edlén Equation for the Refractive Index of Air. Metrologia 31, 315–316, 10.1088/0026-1394/31/4/006 (1994).

[b26] Labib M. . The fluid science laboratory’s microgravity vibration isolation subsystem overview and commissioning update. In SpaceOps Conferences, 10.2514/6.2010-2007 (American Institute of Aeronautics and Astronautics, 2010).

[b27] Microgravity vibration isolation subsystem (MVIS). online at http://www.asc-csa.gc.ca/eng/sciences/mvis.asp., http://www.asc-csa.gc.ca/eng/sciences/mvis.asp.

[b28] Cole G. D., Zhang W., Martin M. J., Ye J. & Aspelmeyer M. Tenfold reduction of Brownian noise in optical interferometry. Nature Photonics 7, 644–650, 10.1038/NPHOTON.2013.174 (2013).

[b29] Bober M. . Strontium optical lattice clocks for practical realization of the metre and secondary representation of the second. Measurement Science and Technology 26, 075201, 10.1088/0957-0233/26/7/075201 (2015).

[b30] Baillard X. . Interference-filter-stabilized external-cavity diode lasers. Optics Communications 266, 609–613, 10.1016/j.optcom.2006.05.011 (2006).

[b31] Millo J. . Ultrastable lasers based on vibration insensitive cavities. Physical Review A–Atomic, Molecular, and Optical Physics 79, 1–7, 10.1103/PhysRevA.79.053829 (2009).

[b32] Thompson R. . A flight-like optical reference cavity for GRACE follow-on laser frequency stabilization. Proceedings of the IEEE International Frequency Control Symposium and Exposition, 10.1109/FCS.2011.5977873 (2011).

[b33] Legero T., Kessler T. & Sterr U. Tuning the Thermal Expansion Properties of Optical Reference Cavities with Fused Silica Mirrors. Journal of the Optical Society of America B 27, 914–919, 10.1364/JOSAB.27.000914 (2010).

